# Antimicrobial peptide LL37 is potent against non-growing *Escherichia coli* cells despite a slower action rate

**DOI:** 10.1128/msphere.00211-24

**Published:** 2024-12-23

**Authors:** Salimeh Mohammadi, Derek Saucedo, Sattar Taheri-Araghi

**Affiliations:** 1 Department of Physics and Astronomy, California State University, Northridge, California, USA; University of Wyoming, Laramie, Wyoming, USA

**Keywords:** antimicrobial peptides, *Escherichia coli*, non-growing cells, persistence

## Abstract

Antimicrobial peptides (AMPs) have long been considered as potential agents against non-growing, dormant cells due to their membrane-targeted action, which is largely independent of the cell’s growth state. However, the relationship between the action of AMPs and the physiological state of their target cells has been unclear, with recent reports offering conflicting views on the efficacy of AMPs against bacteria in a stationary phase. In this study, we employ single-cell approaches combined with population-level experiments to examine the action of human LL37 peptides against *Escherichia coli* cells in different growth phases. Time-lapse, single-cell data from our experiments reveal that LL37 peptides act faster on large, dividing cells than on small, newborn cells. We extend this investigation to non-growing *E. coli* cells in a stationary phase, where we observe that the action of LL37 peptides is slower on non-growing cells compared to exponentially growing cells. This slower action rate is, however, not mirrored in the minimum bactericidal concentration (MBC) measurements. Notably, we find that the MBC for non-growing cells is lower than for exponentially growing cells, indicating that, given sufficient time, LL37 peptides exhibit strong potency against non-growing cells. We propose that the enhanced potency of LL37 peptides against non-growing cells, despite their slower action, can be attributed to continuous absorption of AMPs on the cell membrane over time.

**IMPORTANCE:**

Antibiotic treatments can fail because of the regrowth of a bacterial subpopulation that resumes proliferation once the treatment ceases. This resurgence is primarily driven by non-growing, dormant bacterial cells that withstand the action of antibiotics without developing resistance. In this study, we explore the potency of the human antimicrobial peptide LL37 against non-growing *Escherichia coli* cells. Our findings reveal that despite a slower initial action, LL37 peptides, given sufficient time, demonstrate strong efficacy against non-growing cells. These insights suggest a potential role of antimicrobial peptides in combating persistent bacterial infections by targeting the non-growing cells.

## INTRODUCTION

A major challenge in medicine is the failure of antibiotic treatments due to the regrowth of “persister” cells. These cells, often in a dormant or non-growing state, have the remarkable ability to restart proliferation even after a full course of antibiotic treatment (1–4). Their survival hinges on their phenotypic heterogeneity rather than on the development of genetic resistance (1–3, 5–8). Since dormant cells have slow metabolic activity, they can outlive the action of antibiotics that target pathways associated with bacterial growth and cell-cycle progression (1, 2, 9). This survival strategy signals a pressing need to identify and develop antibiotics that can effectively combat non-growing cells. Antimicrobial peptides (AMPs) have emerged as potentially powerful tools in this fight, with their action considered to be effective against dormant cells (10–12).

AMPs attack bacteria through an intricate physics-based mechanism, utilizing a combination of electrostatic and hydrophobic forces (10, 11). AMPs possess amphiphilic structures, meaning they have both polar, hydrophilic side chains, typically carrying some positively charged residues, and hydrophobic side chains that allow them to interact with non-polar molecules. Such a structure enables AMPs to first bind electrostatically to bacterial membranes and then penetrate the lipid area of the membranes via the hydrophobic side chains (13–19). Penetration of AMPs into the membrane structure ultimately creates trans-membrane pores and compromises the integrity of the target cells. While AMPs are also believed to interact with intracellular compartments, membrane interactions are viewed as a crucial first step in the bactericidal process (20, 21).

However, the relationship between AMPs’ efficacy and the physiological state of target cells remains complex and not fully understood. Factors such as cell-cycle progression, cell age, and cell size appear to influence AMP action, though the specifics are still unknown. Studies at both the single-cell and population levels indicate that AMPs’ activity varies with the growth stage and condition of bacterial cells (22, 23). Single-cell experiments suggest that AMPs act more slowly and are less effective against bacteria in the stationary phase (22, 23). On the other hand, population-level studies have supported the traditional view that AMPs remain effective against non-growing cells, likely due to their membrane-lysing capabilities (24, 25). Understanding the interaction between AMPs and non-growing cells is crucial for advancing treatments for infections that involve dormant, persistent bacteria.

In this work, we employ various single-cell techniques and population-level measurements to systematically investigate the action of LL37 peptides against non-growing *Escherichia coli* cells. We start by measuring how the cell’s growth and size influence the action of LL37 peptides. We find that the susceptibility of *E. coli* cells to dye-tagged LL37 peptides increases as the bacterial cells grow larger and approach the stage of cell division.

Next, we utilize a method to induce a controlled starvation state in *E. coli* cells by depleting nutrients from the cellular microenvironment for a specific period of time, which allows us to study the action of LL37 peptides on non-growing cells. During this nutrient depletion phase, both growth and cell division slows down significantly. Interestingly, we observe that the rate of action of LL37 peptides on individual *E. coli* cells decreases, lagging behind the rate observed on rapidly growing cells.

Yet, this slowdown in action does not translate directly to the minimum bactericidal concentration (MBC) of LL37 peptides. Unexpectedly, we measure that the MBC of LL37 peptides is lower in nutrient-deprived cultures. This indicates that, while their action is slower, LL37 peptides show increased potency against non-growing cells compared to their growing counterparts. This finding sheds new light on the potential therapeutic application of AMPs as antibiotics, especially when targeting persistent infections caused by non-growing, dormant bacteria.

## MATERIALS AND METHODS

### Strain and growth conditions

We conducted all experiments with a non-motile derivative (ΔmotA) of an *Escherichia coli* K12 strain, NCM3722. This strain was previously constructed and validated by Kustu and Jun labs (26, 27). We used a rich defined medium (RDM), formulated by Neidhardt (28) based on 3-(N-morpholino)propanesulfonic acid (MOPS) buffer, which enabled an average doubling time of approximately 23 minutes for the strain at 37°C. This medium is commercially available from Teknova Inc.

### Cell culture preparation

We prepared cell cultures in four stages: seed culture, pre-culture, experimental culture (exponential phase), and starvation culture, where applicable.

For the seed culture, we inoculated an isolated colony from a lysogeny broth (LB) agar plate into 3 mL of RDM in a culture tube. The culture was then incubated, shaking at 37°C overnight. These colonies were grown from a −80°C glycerol stock streaked on the plate and incubated at 37°C for 16–24 hours. The LB plates were stored at 4°C after the initial colony growth for no more than 2 weeks.

For the pre-culture stage, the seed culture was diluted 1,000-fold in the same growth medium following overnight growth and then incubated in a 37°C water bath shaker until it reached the mid-exponential phase.

In the experimental culture phase, cells were harvested at an optical density (OD_600_) of 0.2 and subsequently diluted to an OD_600_ of 0.02 for microscopy and OD_600_ of 0.002 MBC measurement experiments.

In the starvation experiments, 1 mL of the culture was concentrated and washed twice in a buffer containing no nutrients. The culture was then diluted to an OD_600_ of 0.002 to avoid any potential inoculum effect (29–32) and incubated for 3 hours to transition to the stationary phase.

### Antimicrobial peptides

We used the antimicrobial peptide LL37 (AnaSpec, CA, USA) in this study. The peptide, supplied as a dry powder in vials, was reconstituted to 400 mL stocks in autoclaved double-distilled water and stored at –20°C. The net peptide content of the product was 75%, as determined by the manufacturer’s elemental analysis of carbon, hydrogen, and nitrogen. All the supplies used in the experiment, such as pipette tips, micro-centrifuge tubes, and microplates were rated as low protein binding to minimize protein/peptide binding to the experimental supplies.

### Live-cell microscopy

We monitored individual cells under the influence of LL37 peptides using live microscopy. This procedure was similar to the one outlined in reference (29). Individual *E. coli* cells were immobilized on agarose pads (5% agarose gel) for microscopy. Patterned channels in agarose gel were used to distribute cells under the agarose pad in a housing that reduced evaporation, allowing long-term microscopy at 37°C (33, 34). Multiple fields of view from the same sample were studied in each experiment with an interval of 1 minute between time-lapse images.

### Differentiating live and dead cells using BacLight fluorescent stain kit

We used the Live/Dead BacLight fluorescent stain kit (Invitrogen, CA, USA) to distinguish live and dead *E. coli* cells in microscopy experiments. The kit includes two nucleic acid stains: propidium iodide (PI) for dead cells, and SYTO9 for live cells. Once incubated with the culture, the mixture was transferred under an agarose gel pad for time-lapse microscopy. The gel pad contained a lethal dosage of LL37 peptides and a 1:1 mixture of both staining components.

### Determining the minimum bactericidal concentration of LL37 peptides

We determined the MBC of LL37 peptides by assessing the viability of *E. coli* cells following exposure to varying concentrations of LL37. Cultures were prepared as outlined in the Cell Culture Preparation section, diluted to an OD_600_ of 0.002 for both exponential and stationary phase cells. For stationary phase cultures, dilution was performed based on the OD measurement of the culture prior to transitioning to the stationary phase.

For the MBC assay, *E. coli* cultures (both exponential and stationary phases) were exposed to LL37 in a 96-well plate with eight replicates per condition. We repeated the experiment with two biological samples across two 96-well plates. The LL37 concentrations ranged from 0.3 to 1.125 µM (in increments of 0.075 µM) for exponentially growing cells and from 0.075 to 0.900 µM (in increments of 0.075 µM) for stationary phase cells.

After incubating the cells with LL37 for 3 hours at 37°C, 10 µL of culture from each well was transferred to a new 96-well plate, with each well containing LB agar. After 16–24 hours of incubation at 37°C, colony growth in each well was used as a marker of viability. The lowest concentration of LL37 that resulted in no growth (complete inhibition) was designated as the MBC.

## RESULTS

### Susceptibility of *E. coli* cells to LL37 peptides varies with cells’ lifecycle stage

In order to assess bacterial growth inhibition at a single-cell resolution and quantify the distribution and penetration of antimicrobial peptides within target cells, we previously conducted live microscopy on *E. coli* cells using a dye-tagged version of LL37 peptide, 5-FAM-LC-LL37 (29). We harvested cells from a mid-exponential phase culture and brought them in a live microscopy setting where the cells were immobilized under an agarose gel pad infused with both growth media (RDM) and a lethal concentration (10 µM) of 5-FAM-LC-LL37 peptides. Time-lapse data from both phase contrast and fluorescence microscopy revealed rapid absorption and sequestration of a significant quantity of peptides within the target cells upon their death (29).

**Fig 1:**
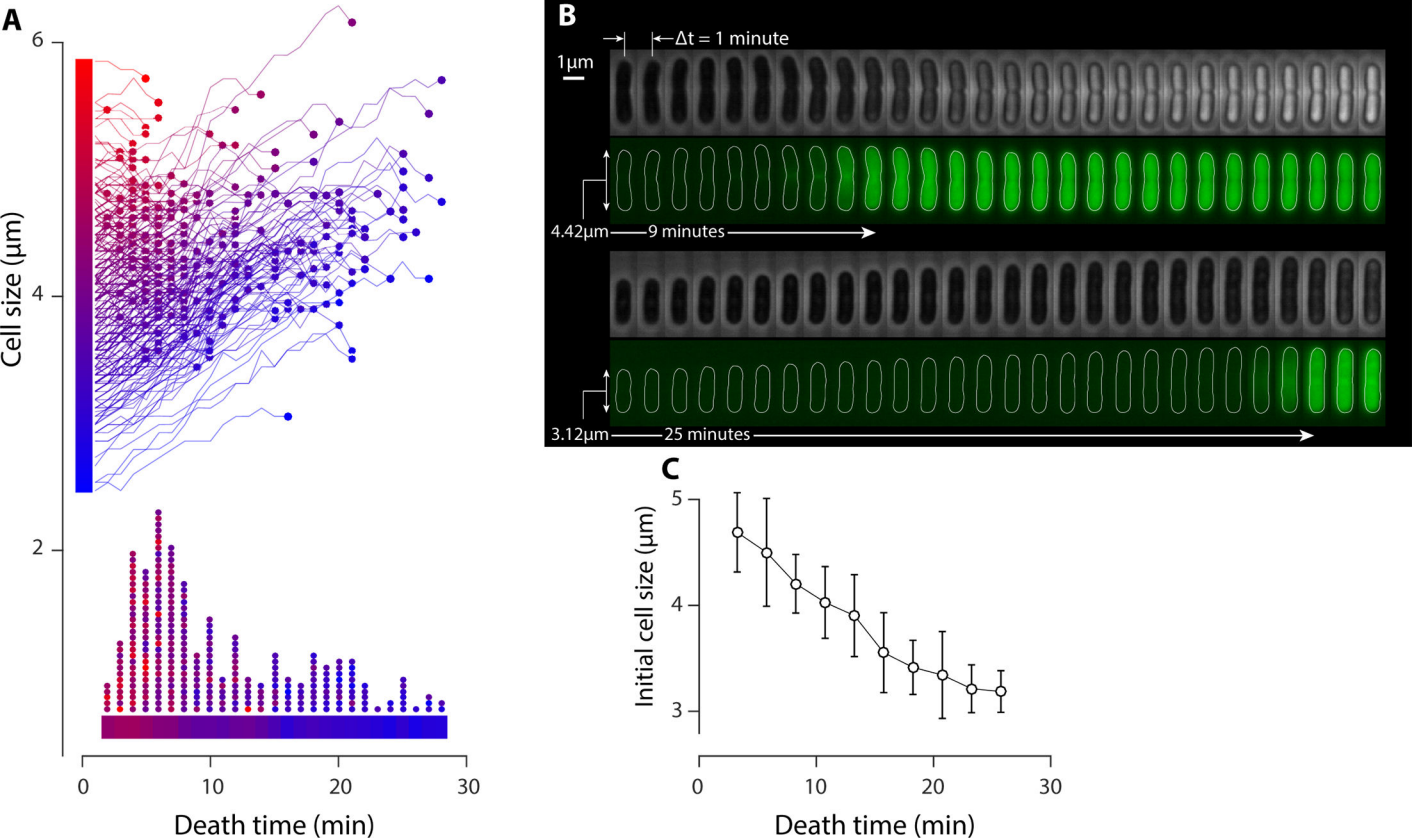
Single-cell experiments reveal a correlation between cell size and susceptibility of *E. coli* cells to LL37 peptides. (**A**) Growth trajectories of individual *E. coli* cells prior to cell death by a lethal dosage (10 µM) of 5-FAM-LC-LL37 peptides. Curves are color-coded based on the initial cell size: the blue to red gradient indicates small to large cells as denoted by the vertical bar adjacent to the *y*-axis. The circle at each curve’s end represents the time of cell death. The histogram of death times at the bottom of the panel indicates that smaller cells (blue) die later than larger cells (red). (**B**) Sample phase-contrast and fluorescent images of a large and a small *E. coli* cell. Cell death is marked by a swift translocation of peptides (green signal) into the cells. The larger cell dies after 9 minutes, while the smaller cell survives until 25 minutes. (**C**) The negative correlation between initial cell size and death time demonstrates that larger cells are more susceptible to LL37 than smaller cells. This panel is constructed based on data from panel A.

In the present study, we examine the correlation between cell size and the timing of cell death by analyzing the growth trajectory of each individual cell prior to its death. Here, the moment of death is defined by the time of peptide translocation into the target cells. Figure 1A presents the size evolution of individual cells, with each curve color-coded based on the cell length at the onset of the experiment: blue shades correspond to smaller cells and red shades to larger cells. Located at the bottom of Fig. 1A, the histogram displays the probability distribution of death time, with the horizontal bar representing death times, color-coded based on the average initial cell size. Both the histogram and color gradient suggest a negative correlation between cell length and death time: larger cells tend to die earlier, followed by smaller cells that die later. Figure 1B depicts samples of both a small (3.12 µm) and large (4.42 µm) cell. Despite differing death times (9 and 25 minutes, respectively), post-death cell sizes are relatively similar. This suggests that smaller cells continue to grow until they become susceptible to the action of LL37 peptides. This negative correlation between susceptibility and cell size is further depicted in Fig. 1C, based on the data presented in Fig. 1A.

The correlation between LL37 peptide activity and cell length raises questions about whether the efficacy of the LL37 peptide can be influenced by sub-optimal physiological conditions, specifically those in which the growth and cell-division cycle is slowed down. To answer these questions, we designed experiments to test and quantify the activity of LL37 peptides against *E. coli* cells in different physiological states.

### Nutrient deprivation temporarily halts bacterial growth without impacting cell viability

To explore the relationship between the physiological state of the target cells and their susceptibility to LL37 peptides, we devised and tested a controlled starvation condition for *E. coli* cells. Our aim was to effectively suppress growth and cell division without compromising cell viability. This was achieved by using the buffer component of the growth media (rich defined media as detailed in the supplemental material, section A, at https://doi.org/10.5061/dryad.x3ffbg7vs) without including any carbon, nitrogen, sugar, or supplements. When inoculated in this buffer, the cells displayed no detectable population growth using a spectrophotometer (Genesys 2000, Fisher Scientific).

**Fig 2:**
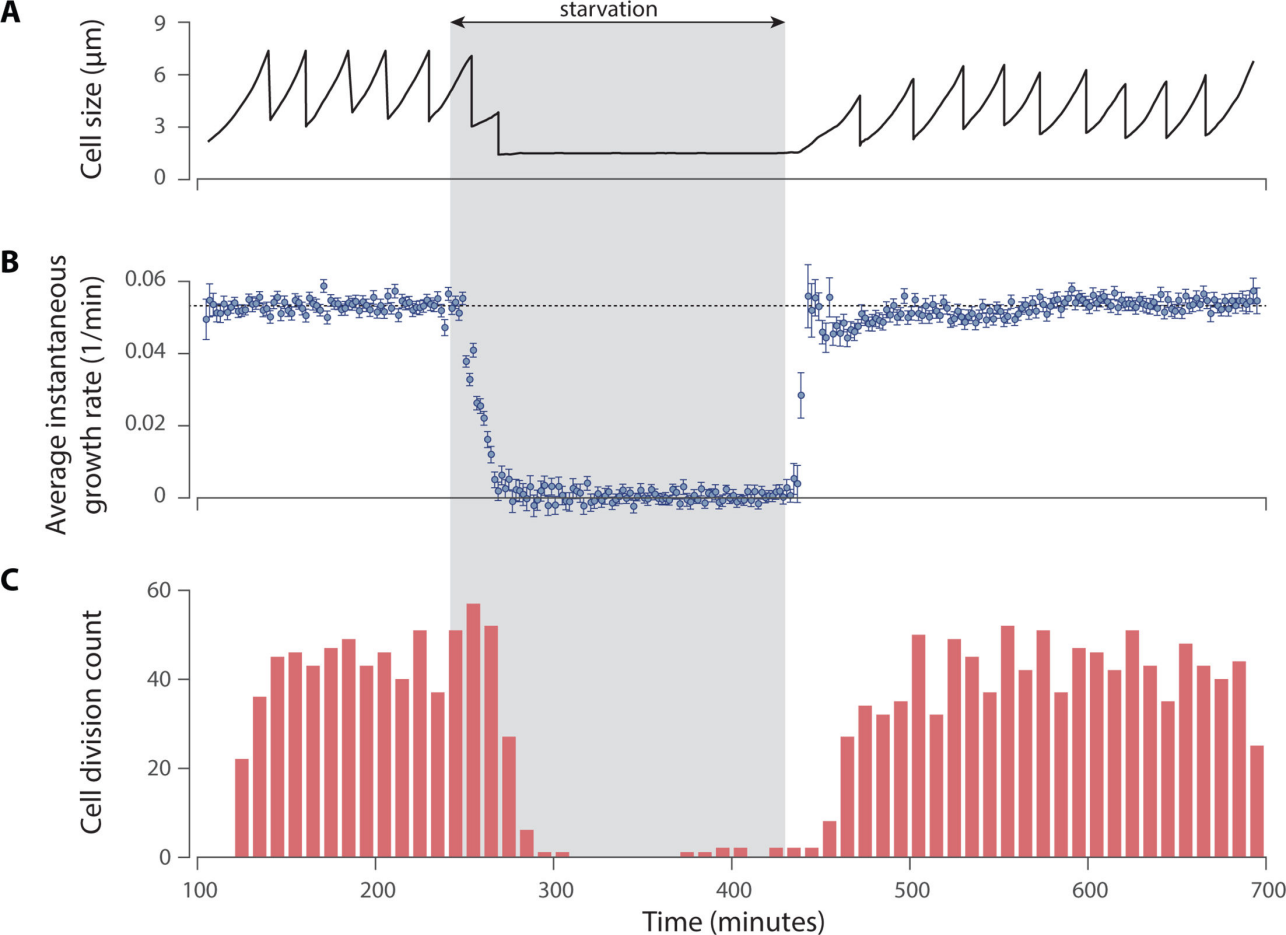
Growth inhibition and viability of *E. coli* cells under nutrient-deprived conditions. (**A**) The growth and division of a sample cell illustrate that growth halts after two cell divisions during the starvation period but rapidly resumes once the growth media are reintroduced. (**B**) The average instantaneous growth rate (elongation rate) of 212 mother cells measured in 2-minute intervals. The data show a rapid decrease in growth rate to zero during the starvation period, with a recovery to normal rates once the growth media are replenished. (**C**) Cell division counts of 212 mother cells recorded in 10-minute intervals. The data show that cell division halts after a brief delay during the starvation period and resumes, also after a delay, once the growth media are reintroduced.

To monitor the transition of cells from the exponential phase to the buffer, we used the microfluidic “mother machine.” This device enables continuous and rapid environmental control along with single-cell resolution microscopy. Approximately 400 individual cells over tens of generations were monitored as they transitioned from RDM to the buffer and back. Specifically, cells were initially grown in RDM within the mother machine for approximately 10 hours, then we switched to the buffer for a duration of 3 hours. The rapid infusion of the media into the microfluidics system allows us to make a swift transition to the nutrient-deprived buffer. We observed that after switching the media to the buffer, the elongation rate of individual cells quickly dropped to zero, and the division rate followed after roughly a 60-minute delay (Fig. 2A and B; Video S1 at https://doi.org/10.5061/dryad.x3ffbg7vs). Over the 3-hour buffer period, growth was largely arrested.

Finally, we switched back to RDM to assess cell viability. We observed that all cells resumed growth shortly after the infusion of RDM, with the cell division rate recovering after a brief delay (Fig. 2C; Video S1 at https://doi.org/10.5061/dryad.x3ffbg7vs). In general, the loss of viability in nutrient-deprived buffers has been reported to occur very slowly, typically following a prolonged lag phase of several hours (35, 36). Therefore, consistent with our observations, it can be reasonably concluded that no significant viability loss due to starvation occurred during the 3-hour buffer exposure period.

In subsequent parts of this study, this transition from growth media to the buffer for 3 hours was used as a method to induce a stationary phase in *E. coli* cells. This provided a platform to investigate the action of LL37 peptides on non-growing cells. Note that the buffer composition matches the ionic strength of the original RDM, ensuring that the absence of growth was solely due to the lack of carbon and nitrogen sources rather than any changes in salt concentration. This consistency in the ionic environment is critical, as fluctuations in ionic strength could alter the electrostatic interactions between LL37 peptides and the bacterial membrane, potentially influencing the peptide’s efficacy.

### Single-cell data indicate prolonged tolerance of non-growing cells to LL37 peptide

To better understand the impact of AMPs on non-growing cells, we conducted similar single-cell experiments with 5-FAM-LC-LL37 peptide, as presented in Fig. 1, on non-growing cells. Specifically, we harvested *E. coli* cells from a culture in the mid-log phase and subjected them to a 3-hour nutrient deprivation period in the buffer solution. The cells were then placed in a live microscopy setting, immobilized under an agarose gel pad containing the nutrient-free buffer and a lethal dose (10 µM) of 5-FAM-LC-LL37 peptides.

**Fig 3:**
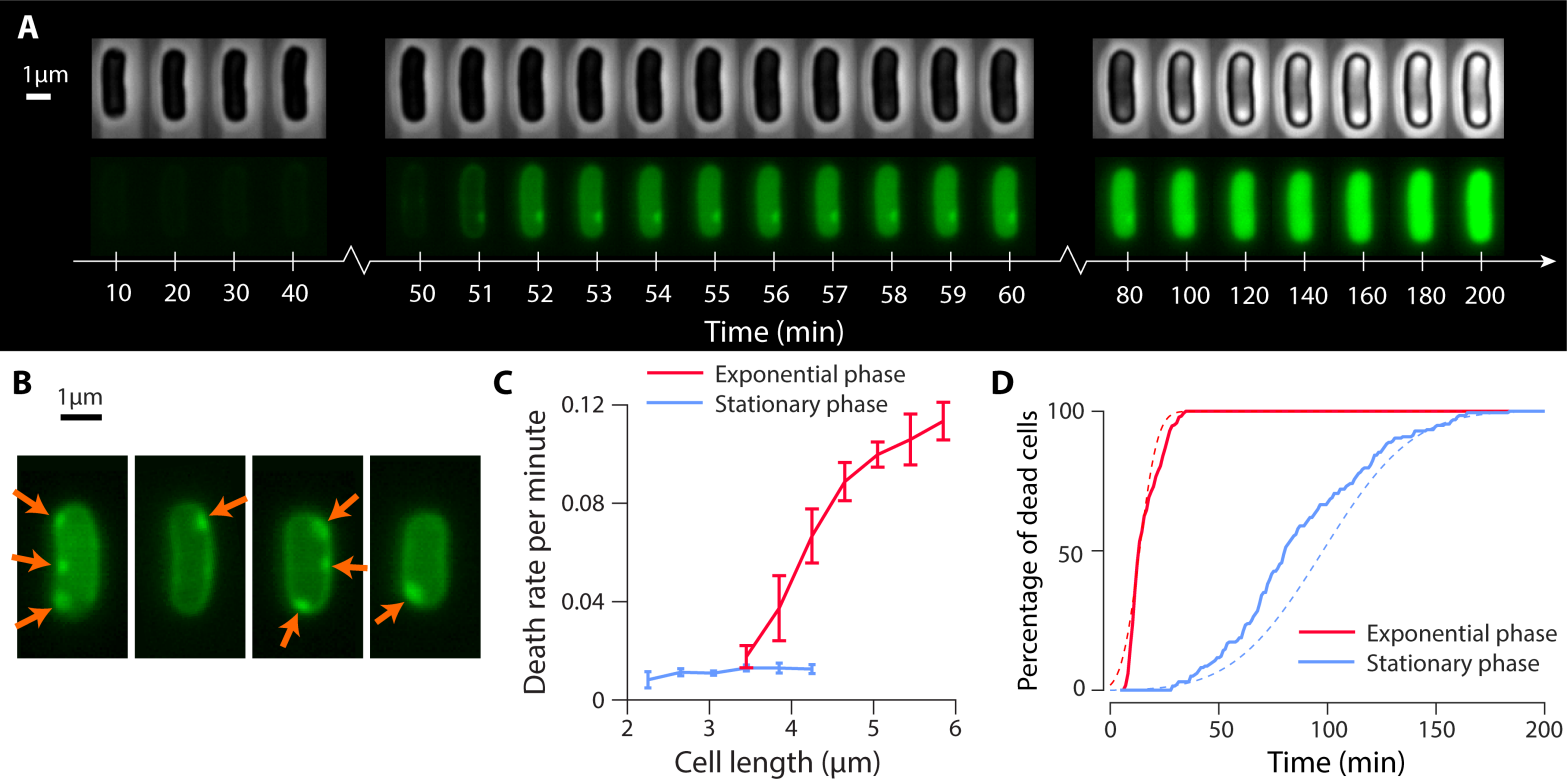
The prolonged tolerance of non-growing *E. coli* cells to 5-FAM-LC-LL37 peptide action. (**A**) Phase contrast and fluorescent time-lapse imaging of a non-growing cell treated with 10 µM of 5-FAM-LC-LL37 peptides. The image sequence shows the slow absorption of AMPs in non-growing cells compared to exponentially growing cells as demonstrated in Fig. 1B. (**B**) Sample of four representative *E. coli* cells, illustrating the initial AMP binding at randomly dispersed locations on the cell surface. (**C**) Variation of death rate per minute as a function of cell size for both exponentially growing and stationary phase cells. Data are binned based on the cell length (*x*-axis), and the error bars show the standard deviation in each bin. (**D**) Plot of the percentage of dead cells (determined by AMP absorption) over time for both exponentially growing and nutrient-starved cells, revealing the slower action of 5-FAM-LC-LL37 peptides on stationary cells. For comparison, the cumulative probability of a normal distribution is depicted by dashed lines.

We observed two significant differences in our microscopy results when compared to those obtained from exponentially growing cells. First, the absorption of peptides into starved cells was notably slower. Figure 3A shows a sample cell where a significant amount of AMP absorption occurs between 50 and 60 minutes after the experiment’s onset. This is followed by slower absorption over an extended period, contrasting starkly with the rapid absorption seen in exponentially growing cells.

Second, the initial accumulation pattern of AMPs on the target cell membrane was different. AMPs bound at random positions on the cell surface, appearing as bright spots around the cell boundary, as shown in Fig. 3B. This differs significantly from the septum-focused binding pattern observed in exponentially growing cells. It should be noted that the puncta observed on the surface of stationary phase cells could potentially be related to surface receptors, although this hypothesis was not explored in the current study.

The physiological state of the target cells visibly influenced the action of the peptides. This was particularly evident when we calculated the death rate (per minute, as inferred by saturation of peptide uptake by cells) and plotted the data by cell length, as shown in Fig. 3C. Non-growing cells’ death rate was considerably smaller than that of exponentially growing cells, which displayed a strong correlation with cell length.

The difference in the kinetics of the action of the antibiotic can also be observed through the percentage (or fraction) of dead cells as a function of time. Figure 3D illustrates how this kinetic varies for non-growing and exponentially growing cells. The solid lines in Fig. 3D represent the experimental data, while the dashed lines are the cumulative probability of a normal distribution based on the mean and standard deviation of rates obtained from the data in Fig. 3C.

Note that non-growing cells demonstrated a slower response, despite being exposed to the same lethal dosage of 5-FAM-LC-LL37 under identical environmental conditions. This suggests that the timescale of AMP action is prolonged primarily due to the growth state of the target cells.

### Assessing tolerance of non-growing versus exponentially growing cells to untagged LL37 peptides using cell viability markers

In another effort to further investigate the susceptibility differences between non-growing and exponentially growing *E. coli* cells to AMPs, we sought to use a method that allows us to use untagged versions of LL37 peptides, thereby mitigating the potential impact that fluorescent tags could have on AMP action.

To this end, we employed the Live/Dead BacLight bacterial viability kit (Life Technologies, CA, USA) to monitor the bactericidal activity of untagged LL37 peptides on individual *E. coli* cells under different growth conditions—stationary or exponential. This kit has two nucleic acid stains: PI, a red-fluorescent dye that penetrates and stains the DNA of bacteria with damaged membranes; and SYTO9, a green-fluorescent dye that stains the DNA of live cells with intact membranes. The difference in spectral properties and permeation capabilities of these stains makes them an ideal choice for investigating the temporal dynamics of cell death through time-lapse microscopy experiments.

One limitation of this method is the delay in the response time of the dyes, which refers to the time required for the dyes to translocate into the cell and bind to nucleic acids before fluorescing. This delay can affect the precise, high-resolution determination of the exact moment of cell death. As a result, the measured timing of cell death may not correspond to the actual moment of cell death but rather a slightly delayed indication. Therefore, the analysis and conclusions drawn in this section are primarily based on variations in the average intensity and timing shifts, which are influenced by the physiological state of the cell.

We prepared the *E. coli* cells in either the exponential or stationary phase (refer to the supplemental material, section B at https://doi.org/10.5061/dryad.x3ffbg7vs) and placed them under an agarose gel pad in a microscopy setting. This pad contained the corresponding media (RDM or buffer), a 5 µM dose of LL37 peptides, and a mixture of PI and SYTO9 stains.

**Fig 4:**
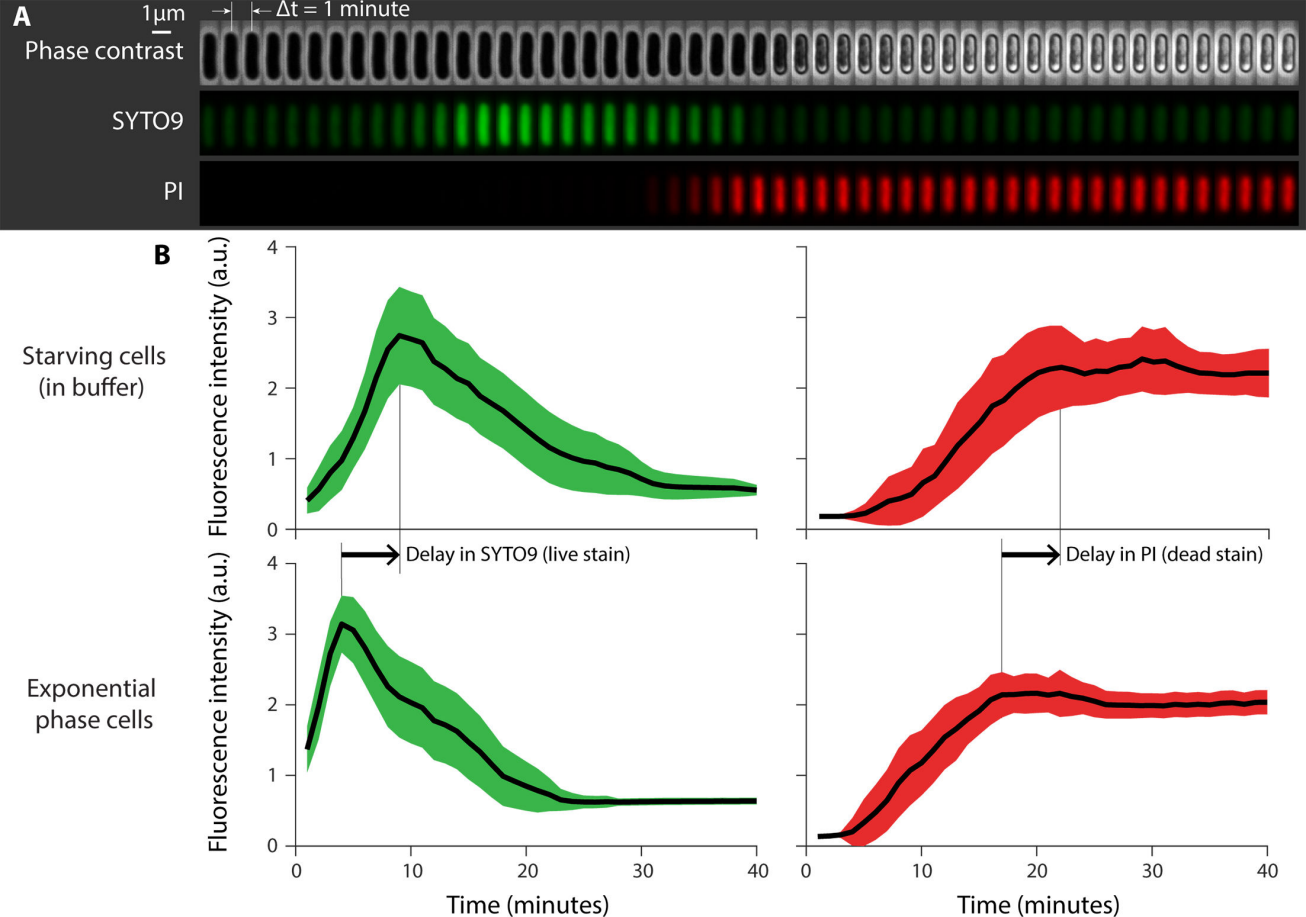
Single-cell microscopy demonstrates prolonged tolerance of non-growing cells to LL37 peptides. (**A**) Time-lapse images of an *E. coli* cell exposed to a lethal dosage (5 µM) of LL37 peptide, with cell viability being tracked using Live/Dead stains. SYTO9 signal (green) dissipates upon cell death, while the PI signal (red) emerges due to increased membrane permeability. (**B**) Fluorescence intensity profiles of SYTO9 (green, left panels) and PI (red, right panels) for 97 nutrient-starved cells (top panels) and 139 exponentially growing cells (bottom panels). Starvation was induced by keeping the cells in a buffer for 3 hours before the microscopy experiment. A comparative analysis of the SYTO9 signal peak and the PI signal plateau reveals a roughly 5-minute delay in the death of nutrient-starved cells. The green and blue shades are 95% confidence intervals.

Figure 4A shows a representative time-lapse outcome of an *E. coli* cell in phase contrast with two fluorescent channels, capturing signals from both SYTO9 and PI. Initially, we observed a gradual increase in the SYTO9 green signal, suggesting a timescale for the complete interaction of SYTO9 with live cells. Following the permeabilization of the cell by LL37 peptides, the red PI signal starts to emerge concurrent with the fading of the green SYTO9 signal.

Figure 4B illustrates the averaged intensity of the stains’ fluorescent signals across an area of 139 exponential phase cells and 97 starvation phase cells. Despite the qualitative similarity in signal changes—indicating that all cells initially are alive and die over the course of the time-lapse imaging due to LL37 peptides—the data reveal a delayed action of LL37 peptides on nutrient-starved cells compared to their exponentially growing counterparts. Specifically, the peak of the average SYTO9 signal (Fig. 4B, left panels) and the plateau of the PI signal (Fig. 4B right panels) both occur approximately 5 minutes later for nutrient-starved cells.

### Reduced minimum bactericidal concentration of LL37 peptides in response to nutrient-starved cells

The extended tolerance of non-growing cells against LL37 peptides prompted us to examine how a bacterial culture in a nutrient-deprived, stationary phase would respond to AMPs. AMPs have long been considered effective agents against non-growing cells (11), and recent studies have supported this claim (24). However, our single-cell observations suggest that this tolerance may be more nuanced. To explore this further, we conducted a comparative study of the MBC of LL37 peptides in nutrient-starved, stationary phase cells versus exponentially growing cells.

To determine the MBC, *E. coli* cultures were prepared as outlined in the Sample Preparation section and diluted to an OD_600_ of 0.002 for both exponential and stationary phase cells. For the stationary phase cultures, dilution was based on the OD measurement of the culture prior to transitioning to the stationary phase. Cultures were then exposed to varying concentrations of LL37 in a 96-well plate, with each condition tested in eight replicates. The LL37 concentrations ranged from 0.3 to 1.5 µM (in increments of 0.075 µM) for exponentially growing cells and from 0.1 to 1.2 µM (in increments of 0.075 µM) for stationary phase cells.

After incubating the cells with LL37 for 3 hours at 37°C, 10 µL of culture from each well was transferred to a new 96-well plate containing LB agar. The plates were incubated for 16–24 hours at 37°C, and colony growth was assessed to determine cell viability. The MBC was defined as the lowest concentration of LL37 that resulted in complete inhibition of colony growth. We repeated the experiment with two biological samples across two 96-well plates for greater precision, ensuring a total of 16 replicates per condition.

**Fig 5:**
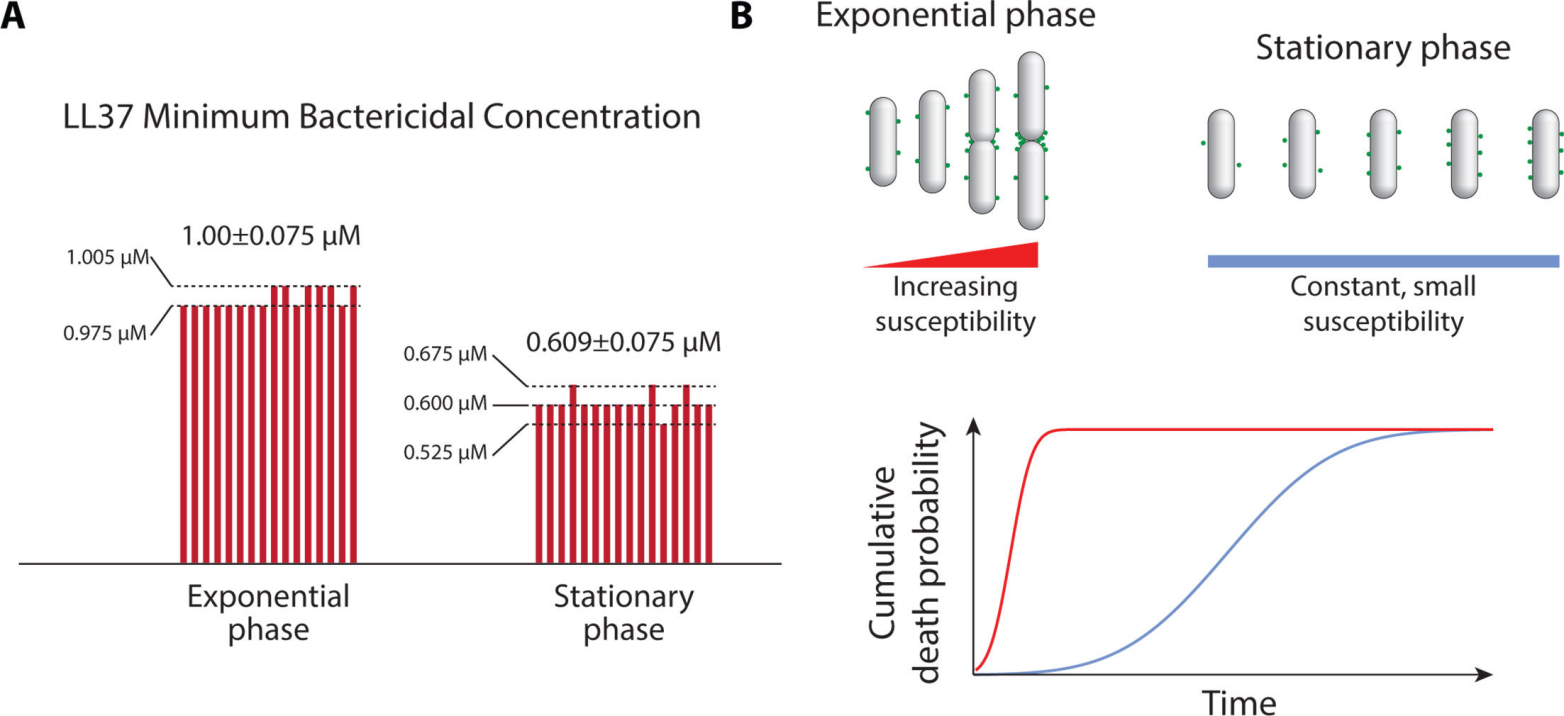
Minimum bactericidal concentration of LL37 peptides and susceptibility models for growing and non-growing *E. coli* cultures. (**A**) Comparison of the MBC of LL37 peptides in exponentially growing and nutrient-starved *E. coli* cultures. The figure presents 16 bars for each condition (two biological samples, with 16 replicates in total per condition). The bar heights represent the MBC values measured for each replicate. Exponentially growing cells had an MBC of 1.00 ± 0.075 µM, while stationary phase cells had a lower MBC of 0.609 ± 0.075 µM. (**B**) A proposed model where the susceptibility of growing cells to AMPs depends on cell age, while the susceptibility of non-growing cells is driven by a slow, continuous, and prolonged absorption of peptides on their membranes.

Contrary to the conventional expectation that a faster killing rate correlates with a lower MBC (37), our results reveal an intriguing deviation from this paradigm. LL37 peptides, although acting more slowly on non-growing cells, have a lower MBC in nutrient-starved *E. coli* cells, compared to exponentially growing cells. Specifically, the MBC for nutrient-starved cells was 0.609 ± 0.075 µM, while for exponentially growing cells, the MBC was 1.00 ± 0.075 µM, as shown in Fig. 5A. This finding highlights the potential of AMPs, particularly LL37, as effective agents against non-growing bacterial cells, despite their slower action.

## DISCUSSION

Antibiotic therapy failures can often be traced back to the decreased susceptibility of dormant or non-growing cells to antibiotics. This reduced susceptibility arises as many antibiotics primarily target growth and cell-cycle-related pathways (1, 6, 38–42). Conversely, antimicrobial peptides are traditionally hypothesized to maintain their efficacy against non-growing cells, given their action on the membrane, which is mostly independent of bacterial growth state (10, 11). In the past few years, however, there were conflicting reports in the literature as to whether AMPs are potent against non-growing cells (23–25).

In this study, we leveraged single-cell and population-level experiments to examine the action of the human antimicrobial peptide LL37 on *E. coli* cells in exponential and stationary phases. Single-cell experiments enabled us to measure the timescale of AMP-induced cell death, while population-level measurements determined the minimum bactericidal concentration of LL37 peptides.

While LL37 peptides do not directly target a specific pathway associated with the cell-cycle progression, they exhibit a nuanced, indirect dependency on cell age. A pioneering study by the Weisshaar lab has indicated a preference for LL37 peptides to bind around the septum of dividing cells. Our single-cell observations illustrate that cells approaching or at the point of cell division exhibit increased susceptibility to LL37 peptides. Given the reduced frequency of cell division in nutrient-poor conditions, the action of LL37 peptides is more prolonged on non-growing, nutrient-starved *E. coli* cells compared to exponentially growing cells.

Interestingly, this difference in action rate is not mirrored in the MBC. Our results show that the MBC of LL37 peptides is lower in cultures of nutrient-starved cells than in those undergoing exponential growth. For these experiments, we intentionally used cultures with identical cell densities to avoid the inoculum effect, a significant factor in AMPs’ action (20, 29, 31). Other recent studies that employed population-level measurements also report similar or decreased MBC values for AMPs against non-growing cells (24).

The lower MBC of LL37 peptides in nutrient-starved cultures contradicts the expectation that a smaller action rate of an antibiotic would correspond to a higher MBC. We propose a model based on the notion that the action of LL37 peptides is initiated by an accumulation of peptides on the membrane up to a certain threshold.

In non-growing cells, however, there is no specific accumulation spot. Nevertheless, continuous absorption and local fluctuations of peptide density on the surface can lead to the emergence of local high-density areas. As time progresses, the probability of such a region reaching the threshold density of AMPs increases. Since the surface area of non-growing cells does not expand over time, a sufficiently long period of exposure to AMPs—even a low dosage of AMPs—can ultimately lead to membrane damage (Fig. 5B).

However, this model calls for a direct, quantitative investigation in future research. The questions addressed in this study carry fundamental importance for the development of peptide antibiotics and their synthetic analogs. In clinical infections, bacteria often inhabit low-nutrient or stationary phase conditions. Consequently, it is imperative to design antibiotic agents capable of efficiently targeting these non-growing cells, thereby enhancing the therapeutic efficacy of antibacterial treatments.
